# Supplementary material for: Individual identification of sika deer (Cervus nippon) using short tandem repeat analysis for investigating illegal carcass disposal in Japan
Source: PLoS One. 2025 Apr 17;20(4):e0321305. doi: 10.1371/journal.pone.0321305 (PMC12005556; doi:10.1371/journal.pone.0321305)
Supplement: Attachment — Submitted filename: Response to Reviews.docx [file pone.0321305.s005.docx]

Thank you for your reviewing our manuscript “Individual identification of sika deer (Cervus nippon) by Short Tandem Repeat (STR) analysis for illegal disposal case in Japan”. I apologize for the length of time it has taken to revise the manuscript due to my health issues. We appreciate your time and effort to improve our manuscript. We would like to address each of the reviewers' comments. We have submitted our manuscript to professional English editing agency before submission.

1. Please ensure that your manuscript meets PLOS ONE's style requirements, including those for file naming.

**Answer:** I had revised according to PLOS ONE’s style requirements.

2. When completing the data availability statement of the submission form, you indicated that you will make your data available on acceptance. We strongly recommend all authors decide on a data sharing plan before acceptance, as the process can be lengthy and hold up publication timelines. Please note that, though access restrictions are acceptable now, your entire data will need to be made freely accessible if your manuscript is accepted for publication. This policy applies to all data except where public deposition would breach compliance with the protocol approved by your research ethics board. If you are unable to adhere to our open data policy, please kindly revise your statement to explain your reasoning and we will seek the editor's input on an exemption. Please be assured that, once you have provided your new statement, the assessment of your exemption will not hold up the peer review process.

**Answer:** I have changed the data availability statement so that we can share entire data freely before acceptance.

3. We note you have included a table to which you do not refer in the text of your manuscript. Please ensure that you refer to Table 1 in your text; if accepted, production will need this reference to link the reader to the Table.

**Answer:** I apologize for the confusion. I have changed Table 7 to Table 1 in Line 164.

4. Please review your reference list to ensure that it is complete and correct. If you have cited papers that have been retracted, please include the rationale for doing so in the manuscript text, or remove these references and replace them with relevant current references. Any changes to the reference list should be mentioned in the rebuttal letter that accompanies your revised manuscript. If you need to cite a retracted article, indicate the article’s retracted status in the References list and also include a citation and full reference for the retraction notice.

**Answer:** I apologize again for the confusion. I have revised the reference number in Table 2, [120] to [33], [121] to [34], and [122] to [35], and reformatted the Reference section.

Additional Editor Comments:

Please correct the table referencing as it is incorrect e.g. Table 1 is in the title but Table 7 is referred to in the text.

**Answer:** I apologize for the confusion. I have changed Table 7 to Table 1 in Line 164.

Use dye name in table 1, not the colour.

**Answer:** I have changed blue to FAM, green to VIC, black to NED and red to PET in Table 1.

Ethics statement – what institution approved this work?

**Answer:** This work was approved by Nippon Veterinary and Life Science University.

From Line 321 - State ranges in brackets = (range x – xx) not as as range (x – xx).

**Answer:** I have changed the brackets to (range x-xx).

From Line 334 – Please correct the bracketing errors

**Answe**r: I have corrected the bracketing error to (CSSM019, TGLA53, ETH10) in Line 338.

Line 441 – An argument is make for low DNA concentration affecting the quality of the STR calls but concentration data is not referred to in the results section. The data needs to be highlighted if this is to form part of the discussion. Please provide or mention this in the results section

**Answer:** I added the median DNA concentration and the range for our PCR analysis in the Result section as “The median DNA concentration yield for the PCR analysis was 98.95 (range 7.5-2025.2) ng/μl (Supporting information)”, Line 274-275 and also provided individual measurements in Supporting information. I have also added “**S2 File. This is the S1 File Concentration of DNA (ng/ul) for PCR analysis in 84 sika deer, 2022, Japan.**” in Line 652-653.

Please make sure the Supp. Data is referred to in the main manuscript text.

**Answer:** I added “Genotyping results for 84 sika deer were described in the Supporting information.” in the Result section, Line 285-286.

Improve figure resolution.

**Answer:** I apologize for low resolution. In order to improve figure resolution, I reduced the number of graphs and presented cases CSSM019 and BM6506 which showed high peaks. I changed the figure title to “**This is the S1 Fig Example of analysis of STR loci and CSSM019 and BM6506 of amplified bovine genotypes Panel 3.1 Kit**” in Line 627-628.

**Comments from Reviewer 1:**

Title:
Acronyms should be avoided in the title. A more refined version could be: "Individual Identification of Sika Deer (Cervus nippon) Using Short Tandem Repeat Analysis for Monitoring Illegal Carcass Disposal in Japan." (This is just a reference you can upgrade better than this)

**Answer:** Thank you for your suggestion. I changed the title to “Individual identification of sika deer (*Cervus nippon*) using short tandem repeat analysis for investigating illegal carcass disposal in Japan” in Line 4 and 5.

Authors affiliation should format as Journal style.

**Answer:** I revised the authors’ affiliation as Journal style.

Abstract is too long, update in single paragraph

**Answer:** I shortened the abstract and updated in a single paragraph.

L29 : cases can change to carcasses (Cases it may confuse) update

**Answer:** I revised to “carcasses” in Line 26.

Introduction:
The introduction is well-written and effectively outlines the study's relevance. To enhance the broader context, it is recommended to add a paragraph discussing the distribution and common behavior of sika deer both locally and regionally, referencing recent studies such as:
Galon, E. M. S. (2023). Molecular epidemiological studies on livestock tick-borne parasitic diseases in the Philippines.
Dhakal, T., Kim, T. S., Kim, S. H., Tiwari, S., Kim, J. Y., Jang, G. S., & Lee, D. H. (2023). Distribution of sika deer (Cervus nippon) and the bioclimatic impact on their habitats in South Korea. Scientific Reports, 13(1), 19040.
Kaji, K., Uno, H., & Iijima, H. (2022). Future Challenges for Research and Management of Sika Deer. In Sika Deer: Life History Plasticity and Management (pp. 615-634). Singapore: Springer Nature Singapore.
Dhakal, T., Jang, G. S., Kim, M., Kim, J. H., Park, J., Lim, S. J., ... & Lee, D. H. (2023). Habitat utilization distribution of sika deer (Cervus nippon). Heliyon, 9(10).

**Answer:** Thank you for providing references. I added a paragraph describing sika deer population in Introduction section “Sika deer (*cervus nippon*) are native to Japan and eastern Asia, but are an invasive species with a global distribution, including 39 countries worldwide[6]. Sika deer live in forests and have a small home range, with males having a larger home range than females[7]. The species range has expanded elsewhere in Japan, causing damage to farmland, forestry and natural vegetation through soil erosion, especially in dense areas[8]. A number of reasons have been discussed for the overpopulation of sika deer in Japan, including reduction in human population, decrease in snow cover period, rapid increase in abandoned farmland, declining hunting population and an ageing population[6, 9].” in Line 58-66.

Material and methods
L 154: The protocol should be properly cited. The mention of "Thermo Fisher Scientific Inc." is vague and should be clarified with appropriate reference.

**Answer:** I apologize for our incomplete explanation. I added “NanoDrop Lite (Thermo Fisher Scientific Inc., Walthman, MA, USA) was used to measure DNA concentration and purity.” in the Method section in Line 155-156.

L175-179: Consider including these details as a note beneath Table 1.

**Answer:** I moved “Adapted from the Bovine Genotypes Panel 3.1 Kit protocol.

Chromosome numbers and allele sizes at which each locus was detected were those for bovine.

The di in the repeat base number indicates that the locus consists of repeats of a two-base repetitive sequence.” as a note beneath Table 1 in Line 178-182.

Statistical Analysis: Ensure that all mathematical notations are formatted in italic style throughout the manuscript.

**Answer:** I formatted all the mathematical notations in italic style throughout the manuscript.

Results:
The tables should be formatted consistently according to the journal’s guidelines.

**Answer:** I formatted all the tables according to the journal’s guidelines.

Numerical data should be presented in a scientific format or limiting values to three decimal places both in the text and in the tables to maintain consistency.

**Answer:** I apologize for the confusion. Except for values that interfere with the interpretation of the result, numerical data are revised to three decimal places both in the text and in the tables.

The figures are of insufficient quality; the data is unreadable. Please provide high-resolution images.

**Answer:** I apologize for the low resolution. In order to improve figure resolution, I reduced the number of graphs and presented cases CSSM019 and BM6506 which showed high peaks. I changed the figure title to “**This is the S1 Fig Example of analysis of STR loci and CSSM019 and BM6506 of amplified bovine genotypes Panel 3.1 Kit**” in Line 627-628.

Discussion and conclusion
While the authors have contextualized their findings within the framework of previous research, the manuscript lacks a discussion of future research directions. It is recommended to include potential avenues for further investigation.

**Answer:** Thank you for your suggestion. I have added “Although previous studies have suggested a decrease in genetic diversity of the two-tailed deer due to bottlenecks, the present study showed that the effect was low, and that haplotype and allele frequencies varied among local populations. Therefore, it was considered necessary to select markers appropriate for the local populations to be tested and to determine the degree of genetic diversity when conducting DNA identification in wildlife forensic veterinary medicine. Although further accumulation of data and expansion of markers with higher discriminatory power are needed in the future, this is the first study to evaluate the usefulness of STR analysis for individual identification for sika deer in Japan. and it is expected to be applied to individual identification in wildlife forensic veterinary medicine. Useful marker sets should be developed, and databases should be constructed for other animal species in Japan as well for the future investigation.” in the Discussion section, Line 444-454.
