# Supplementary material for: Individual identification of sika deer (Cervus nippon) using short tandem repeat analysis for investigating illegal carcass disposal in Japan
Source: PLoS One. 2025 Apr 17;20(4):e0321305. doi: 10.1371/journal.pone.0321305 (PMC12005556; doi:10.1371/journal.pone.0321305)
Supplement: Attachment — Submitted filename: Response to Reviewers.docx [file pone.0321305.s006.docx]

Thank you for reviewing our manuscript “Individual identification of sika deer (*Cervus nippon*) by Short Tandem Repeat (STR) analysis for illegal disposal case in Japan”. We appreciate your time and effort to improve our manuscript, and we apologize that we failed to correct the presentation and typographical errors. We carefully reviewed our manuscript and addressed all the comments from the Academic Editor.

Journal Requirements:

Please review your reference list to ensure that it is complete and correct. If you have cited papers that have been retracted, please include the rationale for doing so in the manuscript text, or remove these references and replace them with relevant current references. Any changes to the reference list should be mentioned in the rebuttal letter that accompanies your revised manuscript. If you need to cite a retracted article, indicate the article’s retracted status in the References list and also include a citation and full reference for the retraction notice.

**Answer**: We have carefully reviewed our references and corrected the reference number in Line 426 and 441.

Additional Editor Comments:

Please revise the text for typographical errors. These must be corrected to meet the presentation standards of PLOS One.

**Answer:** I apologize for the typographical errors. We corrected them to meet the presentation standards of PLOS One.

Table 1: please review the journal standards for annotations to tables

**Answer:** We added footnotes in the Table 1 as follows “^a^Chromosome numbers and allele sizes at which each locus was detected were those for bovine.

^b^The di in the repeat base number indicates that the locus consists of repeats of a two-base repetitive sequence.” in Line 179-182.

”
Line 150: Subsidy not Subsid.

**Answer**: I apologize again for our typographical error. I corrected to “subsidy” in Line 150.

Line 214: Remove the sentence "The PCR was carried out as follows'. No instructions follow.

**Answer**: We removed the sentence “The PCR was carried out as follows” in Line 194. I apologize for the confusion.

Line 390: Italics error for He

**Answer**: We changed to Italic as H*e*.

Line 396: Italics error for He and Ho

Answer: We changed to Italic as H*e* and H*o*.

Abbreviations: If you define an abbreviation in the text e.g. PD, use the abbreviation. Define the term the first time it appears in the paper and use only the abbreviation from then on. If a term is only used once, do not give it an abbreviation.

**Answer**: We have revised all the abbreviations throughout the manuscript, including PD, APD. MP, H*o* and H*e*.

Round all numbers to three decimal places: see line 421, 424 and 425.

**Answer**: I apologize that we did not round up to three decimal places. The difference between those numbers were in the 9^th^, 5^th^ and 10^th^ decimal points and if we round them up in three decimal places, we would not be able to show the difference of the identification performances in the previous studies. Therefore, we included calculation formula as follow; “1-3.00 × 10^-9^ (0.999999997), 1-7.00 × 10^-5^ (0.999993) and 1-4.00 × 10^-10^ (0.99999999996)” in Line 400, 403 and 404.

Line 473: Remove the full stop from this sentence "for sika deer in Japan. and it is expected to be ". It should read "...for sika deer in Japan and it is expected to be...."

**Answer**: We apologize for the typographical error. I changed to “for sika deer in Japan and it is expected to be” in Line 452.
